# Metabolic syndrome and concomitant diabetes mellitus are associated with higher risk of cardiovascular comorbidity in patients with primary glomerular diseases: A retrospective observational study

**DOI:** 10.1002/clc.23388

**Published:** 2020-05-29

**Authors:** Zhiyong Xie, Zhilian Li, Wei Dong, Yuanhan Chen, Ruizhao Li, Yanhua Wu, Ting Lin, Yiming Tao, Huaban Liang, Wenjian Wang, Shuangxin Liu, Zhiming Ye, Wei Shi, Xinling Liang

**Affiliations:** ^1^ The Second School of Clinical Medicine Southern Medical University Guangzhou China; ^2^ Division of Nephrology, Guangdong Provincial People's Hospital Guangdong Academy of Medical Sciences Guangzhou China; ^3^ The Second School of Clinical Medicine, Southern Medical University Guangzhou China

**Keywords:** cardiovascular comorbidity, diabetes mellitus, metabolic syndrome, primary glomerular diseases, risk factor

## Abstract

**Background:**

Metabolic syndrome (MS) and diabetes mellitus (DM) are risk factors for cardiovascular diseases in general population. However, there was a paucity of studies investigating their impact in primary glomerular diseases (PGD).

**Hypothesis:**

MS and concomitant DM are associated with higher risk of cardiovascular comorbidity in PGD.

**Methods:**

In a retrospective observational design, we analyzed 3622 hospitalized adult PGD patients and compared the prevalence of cardiovascular comorbidity in non‐MS, MS with and without DM. Risk factors for cardiovascular comorbidity were identified using univariate and multivariate logistic regression.

**Results:**

Among 3622 PGD patients, 308 (8.5%) cases accompanied with MS, including 180 (5.0%) patients with DM and 128 (3.5%) without DM. One hundred and sixty four (4.5%) cases coexisted with cardiovascular comorbidity. Patients with MS and concomitant DM exhibited a higher prevalence of cardiovascular comorbidity than those without MS stratified by estimated glomerular filtration rate and pathological types. Logistic regression showed that MS and concomitant DM (OR: 2.496, 95% CI: 1.600‐3.894, *P* < .001), older age (OR: 1.060, 95% CI: 1.047‐1.074, *P* < .001), male (OR: 1.536, 95% CI: 1.072‐2.200, *P* = .019), higher level of serum ti (OR: 1.002, 95% CI: 1.001‐1.003, *P* < .001), hyperuricemia (OR: 1.901, 95% CI: 1.327‐2.725, *P* < .001), idiopathic membranous nephropathy (OR: 2.874, 95% CI: 1.244‐6.640, *P* < .001) and focal segmental glomerulosclerosis (OR: 2.906, 95% CI: 1.147‐7.358, *P* < .001) were independently associated with a higher risk for cardiovascular comorbidity.

**Conclusions:**

In PGD patients, MS and concomitant DM are associated with an increased risk for cardiovascular comorbidity. More evidence for the causal link between MS/DM and cardiovascular outcomes is needed to be clarified.

## 1 INTRODUCTION

Metabolic syndrome (MS) was widely prevalent and characterized by a cluster of cardiovascular risk factors, including impaired glucose metabolism, dyslipidemia and hypertension. Previous meta‐analysis had found that MS was associated with a twofold increase in cardiovascular outcomes and a 1.5‐fold increase in all‐cause mortality in general population.^1^ Chronic kidney disease (CKD) was another cardiovascular risk factor, which remained a worldwide public health problem and had dramatically increased. It was estimated that the prevalence of CKD was 8% to 16%^2^ in the world and was 10.8% in China.^3^ Chronic Kidney Disease Prognosis Consortium had found that estimated glomerular filtration rate (eGFR) less than 60 mL/min/1.73 m^2^ and albumin creatine ratio greater than 1.1 mg/mmol (10 mg/g) were independent predictors of all‐cause mortality and cardiovascular mortality.^4^ Previous epidemiological studies had reported an increased prevalence of MS in CKD, which was associated with higher risk for disease progression^.^ ^5^ The overall prevalence of MS was 30.5% in stages 4 and 5 CKD patients.^6^ MS portends a higher CVD risk in CKD population. Primary glomerular disease (PGD), as one of the leading causes of CKD, was considered to increase cardiovascular comorbidity and mortality, especially in those with lower eGFR.^7^ The vast majority of PGD were in early stage of CKD and the prevalence of MS in PGD had not been investigated before. In addition, PGD patients were characterized by glomerular leakage of albuminuria and susceptible to insulin resistance or hyperglycemia, dyslipidemia and hypertension.^8^ Therefore, PGD and MS may share some common metabolic characteristics. However, it remained unclear whether MS was independently associated with higher cardiovascular risk in PGD patients.

Diabetes mellitus (DM) was another independent risk factor for cardiovascular disease and was frequently seen in PGD patients. Among a Chinese CKD cohort study, DM was independently associated with higher prevalence of cardiovascular disease.^9^ However, prior results had not explored whether MS coexisted with DM or not would increase risk for cardiovascular disease in PGD. Proteinuria, renal insufficiency in combination of MS and DM may exert a severer influence on the cardiovascular comorbidity risk. Thus, we aimed to investigate the effect of MS in subjects with and without DM on cardiovascular comorbidity risk in PGD population.

## 2 METHODS

### 2.1 Study design and patients

Five thousand four hundred and two consecutive patients performed kidney biopsy from January 2007 to December 2018 in division of nephrology, Guangdong Provincial People's Hospital were selected for this retrospective observational study. The inclusion criteria were defined as follows: (a) patients were more than 18 years old; (b) patients were diagnosed with pgd. patients diagnosed with secondary glomerular diseases (1558), tubulointerstitial diseases (94), hereditary renal diseases (33) or unclassified cases (95) were excluded. All patients undergoing renal biopsy would be performed serologic detection including antinuclear antibody (ANA), anti‐dsDNA antibody, extractable nuclear antigen antibody (ENA), antineutrophil cytoplasmic antibody (ANCA), anti‐glomerular basement membrane (GBM) antibody, complement C3, complement C4, serum protein electrophoresis (SPE) / serum immunofixation electrophoresis (SIFE), anti‐hepatitis B virus/hepatitis C virus (HBV/HCV), cryoglobulin, tumor markers (such as prostate specific antigen, free prostate specific antigen, and alpha fetoprotein) to screening for the secondary etiology including infectious diseases, rheumatic immune diseases and tumor. Patients would also be performed chest X‐ray, color doppler ultrasound of thyroid gland, liver, gallbladder, pancreas, spleen and gynecological organ (womb and appendix in female) to exclude malignancy. Gastrointestinal endoscopy was performed in patients at high risk of tumor to exclude gastrointestinal tumors. Patients suspected tumor were suggested to perform positron emission tomography/computed tomography (PET/CT). In addition, doctors‐in‐charge would inquiry about medical history including medication history and past history to screen secondary etiologic factors including medicine and infection. PGD was diagnosed based on the serological, radiological and renal pathological data. Finally, 3622 PGD patients were included in this study. All laboratory data and medical records were derived from electronic medical records. The study complied with the declaration of Helsinki and was approved by the Ethics Committee of Guangdong Provincial People's Hospital (No. GDREC2018541H). The committee waived the requirement for informed consent in consideration of retrospective nature of the study, and the data were analyzed anonymously.

### 2.2 Data collection and definitions

Baseline demographic data including age, sex, height, and weight were collected manually from medical record. Laboratory data including serum creatine, serum albumin, 24 hours ours proteinuria, uric acid, hemoglobin, cholesterol (CHOL), triglyceride (TG), high‐density lipoprotein‐cholesterol (HDL‐C) and low‐density lipoprotein‐cholesterol (LDL‐C) were recorded at admission. MS was defined as fulfilling at least two of the following criteria^10^: (1) high‐blood pressure (≥130/85 mmHg) or current use of antihypertensive medication; (2) high TG (≥1.7 mmol/L); (3) low HDL‐C (<1.03 mmol/L in men and <1.29 mmol/L in women); (4) high fasting blood glucose level [≥100 mg/dL (5.6 mmol/L)] or previously diagnosed DM. MS patients were categorized as having DM or not based on the International Diabetes Federation (IDF) definition.^11^ Cardiovascular comorbidity was defined as a documented diagnosis of congestive heart failure (NYHA class II or greater) or ischemic heart disease caused by coronary artery disease with or without myocardial infarction or clinical manifestations of peripheral vasculopathy or cerebrovascular disease (stroke, transischemic attack).^12^ Body mass index (BMI, kg/m^2^) was calculated as weight in kilograms divided by height in meters squared. We used the formula of the Chronic Kidney Disease Epidemiology Collaboration (CKD‐EPI) to calculate the baseline eGFR.^13^ Histological classification of kidney disease was comprised of PGD, secondary glomerular diseases, tubulointerstitial diseases, hereditary renal diseases and unclassified cases according to “Revised Protocol for the Histological Typing of Glomerulopathy” issued by the World Health Organization (WHO) in 1995^.^ ^14^ PGD included IgA nephropathy (IgAN), idiopathic membranous nephropathy (IMN), minimal change disease (MCD), focal segmental glomerulosclerosis (FSGS), mesangial proliferative glomerulonephritis (MsPGN), membranoproliferative glomerulonephritis type 1 (MPGN I), dense deposit disease (DDD), MPGN III, C3 glomerulonephritis (C3 GN), endocapillary proliferative glomerulonephritis, crescentic glomerulonephritis and others. Hypertension, hyperuricemia, hyperlipidemia, anemia, and cardiovascular comorbidity were identified based on reported history and discharge diagnosis. Hypertension was defined as a systolic blood pressure 140 mmHg or more, diastolic blood pressure 90 mmHg or more, or treatment with antihypertensive medicine. Hyperlipidemia was defined as blood triglyceride ≥1.70 mmol/L and/or total cholesterols ≥5.18 mmol/L. Hyperuricemia was defined as a serum uric acid level ≥420 μmol in males or ≥360 μmol in females or based on the use of urate‐lowering medications. Anemia was defined as hemoglobin <120 g/L in women and <130 g/L in men. Microscopic hematuria was defined as three or more red blood cells per high‐power field on at least two different occasions.

### 2.3 Statistical analysis

All data were analyzed using SPSS statistical software for Windows, version 23.0 (SPSS, Inc., Chicago, IL). Measurement data with a normal distribution are expressed as the mean ± SD, and differences among groups were compared using one‐way ANOVA followed by a Bonferroni post hoc test. The measurement data discordant with a normal distribution are expressed as medians (25th, 75th percentiles), and differences between two groups were compared using the Mann‐Whitney *U* test. Categorical data are expressed as cases (percentages), and differences between groups were compared using the *χ* ^2^ test. Logistic regressions were performed to analyze risk factors for cardiovascular comorbidity, and odds ratios (ORs) and 95% confidential intervals (95% CIs) were calculated. The adjusted variables were selected into the multivariate regression model (forward selection stepwise; *P* < .05 criterion for variable retention) based on the univariate regression analyses. Two‐tailed tests were used for all comparisons, and *P* < .05 was considered to be statistically significant.

## 3 RESULTS

### 3.1 Clinical characteristics of PGD patients with and without MS

Among 3622 consecutive PGD patients, 308 patients (8.5%) were diagnosed with MS, including 180 (5.0%) patients with DM and 128 (3.5%) without DM. Compared with non‐MS, MS patients exhibited higher levels of BMI, serum creatine, 24 hours proteinuria, uric acid, TG and lower levels of eGFR and HDL‐C. In addition, MS patients were elder and coexisted with higher proportion of hypertension, hyperlipidemia, and hyperuricemia. Compared to MS alone, age, proteinuria, uric acid, TG and proportion of hypertension, hyperuricemia, and hyperlipidemia were significantly higher in subjects with MS and concomitant DM (Table 1).

**TABLE 1:** Clinical baseline characteristics of primary glomerular diseases patients with and without metabolic syndrome

	Total (n = 3622)	Non‐MS (n = 3314)	MS		*P* value
			MS without DM (n = 128)	MS with DM (n = 180)	
Male (n, %)	1904 (52.6)	1731 (52.2)	68 (53.1)	105 (58.3)	.277
Age (years)	40.9 ± 15.3	39.9 ± 15.0	46.2 ± 13.6*	57.0 ± 11.3^&^ ^,^ ^#^	<.001
BMI (kg/m^2^)	23.5 ± 3.9	23.3 ± 4.0	25.0 ± 3.2*	25.1 ± 3.6^#^	<.001
Serum creatine (μmol/L)	96.0 (70.0, 150.0)	94.6 (69.2, 144.0)	134.1 (88.3, 225.8)*	112.6 (76.7, 199.9)^#^	<.001
eGFR (ml/min/1.73 m^2^)	73.4 ± 36.3	75.1 ± 36.2	52.0 ± 30.0*	57.0 ± 33.1^#^	<.001
eGFR stratification					
≥90 mL/min/1.73 m^2^	1375 (38.0)	1313 (39.6)	19 (14.8)*	43 (23.9)^#^	<.001
60‐89 mL/min/1.73 m^2^	894 (24.6)	824 (24.9)	29 (22.6)	41 (22.8)	.707
30‐59 mL/min/1.73 m^2^	786 (21.7)	693 (20.9)	45 (35.2)*	48 (26.6)	<.001
15‐29 mL/min/1.73 m^2^	376 (10.4)	326 (9.8)	23 (18.0)*	27 (15.0)^#^	.001
<15 mL/min/1.73 m^2^	191 (5.3)	158 (4.8)	12 (9.4)*	21 (11.7)^#^	<.001
Serum albumin (g/L)	27.8 ± 10.4	27.7 ± 10.4	33.3 ± 8.9*	26.5 ± 9.5^&^	<.001
Proteinuria (g/24 h)	2.4 (0.8, 5.9)	2.3 (0.8, 5.8)	2.3 (1.0, 4.2)^#^	4.2 (1.8, 9.6)^&^	<.001
Uric Acid (μmol/L)	399.4 ± 172.4	394.7 ± 173.6	474.4 ± 137.8*	432.6 ± 155.4^&^ ^,^ ^#^	<.001
Hemoglobin (g/L)	130.0 ± 21.9	130.0 ± 22.0	131.6 ± 20.6	127.9 ± 21.4	0.308
Cholesterol (mmol/L)	6.9 ± 3.5	6.9 ± 3.5	6.5 ± 2.5	7.0 ± 3.3	.376
Triglyceride (mmol/L)	2.4 ± 2.1	2.3 ± 1.9	3.0 ± 1.9*	3.7 ± 3.6^&^ ^,^ ^#^	<.001
HDL‐C (mmol/L)	1.3 ± 0.6	1.3 ± 0.6	1.2 ± 0.5*	1.2 ± 0.5^#^	.02
LDL‐C (mmol/L)	4.0 ± 2.2	4.0 ± 2.3	4.1 ± 1.7	4.1 ± 2.1	.816
Hypertension (n, %)	1226 (33.8)	932 (28.1)	128 (100.0)*	166 (92.2)^&^ ^,^ ^#^	<.001
Hyperuricemia (n, %)	810 (22.4)	669 (20.2)	85 (66.4)*	56 (31.1)^&^ ^,^ ^#^	<.001
Hyperlipidemia (n, %)	320 (8.8)	157 (4.7)	128 (100.0)*	35 (19.4)^&^ ^,^ ^#^	<.001

Abbreviations: BMI, body mass index; DM, diabetes mellitus; eGFR, estimated glomerular filtration rate; HDL‐C, high density lipoprotein‐cholesterol; LDL‐C, low density lipoprotein‐cholesterol; MS, metabolic syndrome.

* *P* < .05: the MS without DM group vs the non‐MS group.

# *P* < .05: the MS with DM group vs the non‐MS group.

& *P* < .05: the MS with DM group vs the MS without DM group.

### 3.2 Clinical characteristics of PGD patients with and without cardiovascular comorbidity

The prevalence of cardiovascular comorbidity was 4.5% (164/3622) in PGD. Compared with non‐cardiovascular comorbidity, more patients with cardiovascular comorbidity were male and elder, and presented with higher levels of serum creatine, 24 hours proteinuria, uric acid, TG and LDL‐C. Additionally, they exhibited lower levels of eGFR, serum albumin and hemoglobin but a higher proportion of hypertension, diabetes, hyperuricemia and hyperlipidemia (Table S1).

### 3.3 Prevalence of MS and cardiovascular comorbidity stratified by eGFR and pathological types

**FIGURE 1:**
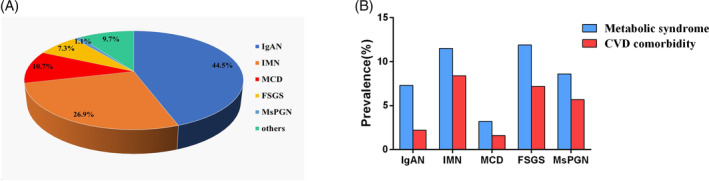
Prevalence of metabolic syndrome and cardiovascular comorbidity by category of pathological types. A, Prevalence of different pathological type in primary glomerular diseases. B, Prevalence of metabolic syndrome and cardiovascular comorbidity in different pathological types. FSGS, focal segmental glomerulosclerosis; IgAN, IgA nephropathy; IMN, idiopathic membranous nephropathy; MCD, minimal change disease; MsPGN, mesangial proliferative glomerulonephritis

The pathological composition of 3622 PGD patients are provided in Figure 1A. IgA predominated in PGD, followed by IMN, MCD, FSGS, and MsPGN, with the prevalence of 44.5%, 26.9%, 10.7%, 7.3%, and 1.1%, respectively. Among 164 cases with cardiovascular comorbidity, 41 were ischemic heart disease, 50 were cerebrovascular disease, 57 were peripheral vasculopathy and 25 were congestive heart failure. Among the five most common PGD, higher prevalence of MS and cardiovascular comorbidity were noted in IMN and FSGS. The prevalence of MS and cardiovascular comorbidity in IMN were significantly higher than IgAN (MS: *χ* ^2^ = 12.794, *P* < .001; cardiovascular comorbidity: *χ* ^2^ = 54.439, *P* < .001) and MCD (MS: *χ* ^2^ = 18.289, *P* < .001; cardiovascular comorbidity: *χ* ^2^ = 20.143, *P* < .001). The prevalence of MS and cardiovascular comorbidity in FSGS were also higher than IgAN (MS: *χ* ^2^ = 9.821, *P* = .002; cardiovascular comorbidity: *χ* ^2^ = 25.177, *P* < .001) and MCD (MS: *χ* ^2^ = 18.091, *P* < .001; cardiovascular comorbidity: *χ* ^2^ = 14.401, *P* < .001). However, there was no difference in the prevalence of MS and cardiovascular comorbidity between IMN, FSGS, and MsPGN (Figure 1B).

### 3.4 Higher prevalence of cardiovascular comorbidity in PGD patients with metabolic syndrome and concomitant diabetes mellitus

**FIGURE 2:**
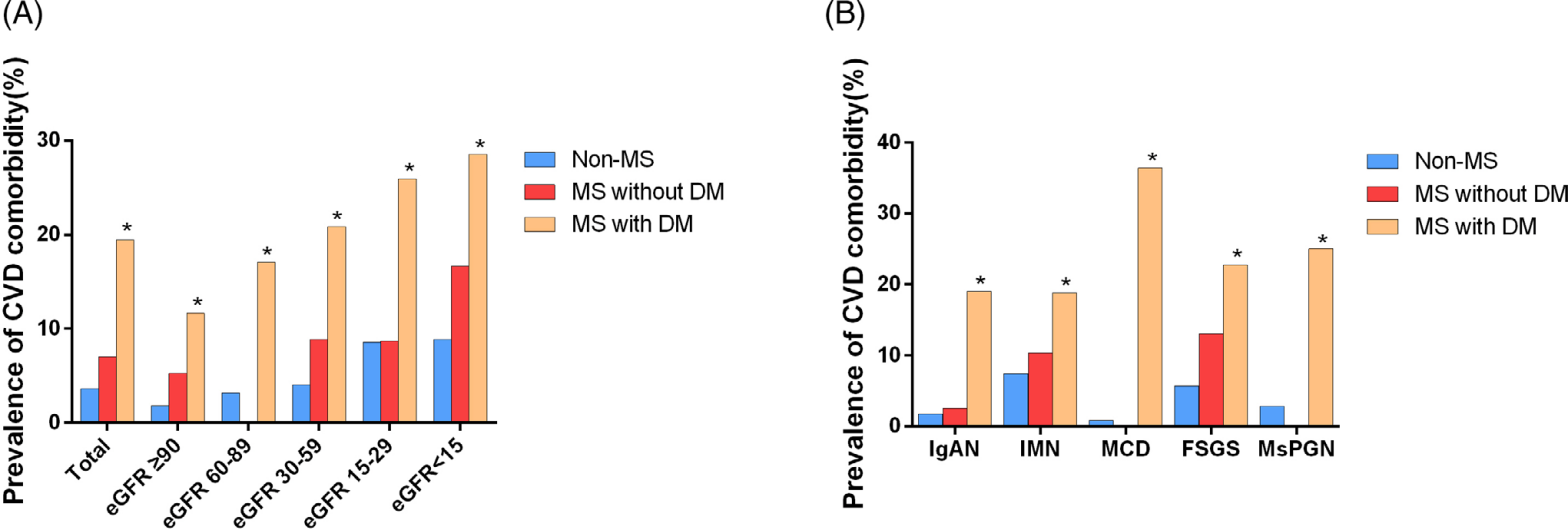
Prevalence of cardiovascular comorbidity in non‐metabolic syndrome, metabolic syndrome with and without diabetes mellitus. A, Prevalence of cardiovascular comorbidity in different levels of eGFR. B, Prevalence of cardiovascular comorbidity in different pathological types. DM, diabetes mellitus; eGFR, estimated glomerular filtration rate; FSGS, focal segmental glomerulosclerosis; IgAN, IgA nephropathy; IMN, idiopathic membranous nephropathy; MCD, minimal change disease; MS, metabolic syndrome; MsPGN, mesangial proliferative glomerulonephritis. CVD, cardiovascular disease. *P < .05, non‐metabolic syndrome vs metabolic syndrome with diabetes mellitus

The prevalence of cardiovascular comorbidity categorized by different levels of eGFR and different pathological types of PGD were shown in Figure 2. Compared with non‐MS, Patients with MS and concomitant DM presented with higher prevalence of cardiovascular comorbidity in different levels of eGFR. However, there was no significant difference in the prevalence of cardiovascular comorbidity between non‐MS and MS without DM (Figure 2A). In different pathological types of PGD, patients with MS and concomitant DM all had higher prevalence of cardiovascular comorbidity than those without MS, and the difference was also statistically significant (Figure 2B).

### 3.5 Metabolic syndrome and concomitant diabetes mellitus were associated with an increased risk for cardiovascular comorbidity in primary glomerular diseases

**FIGURE 3:**
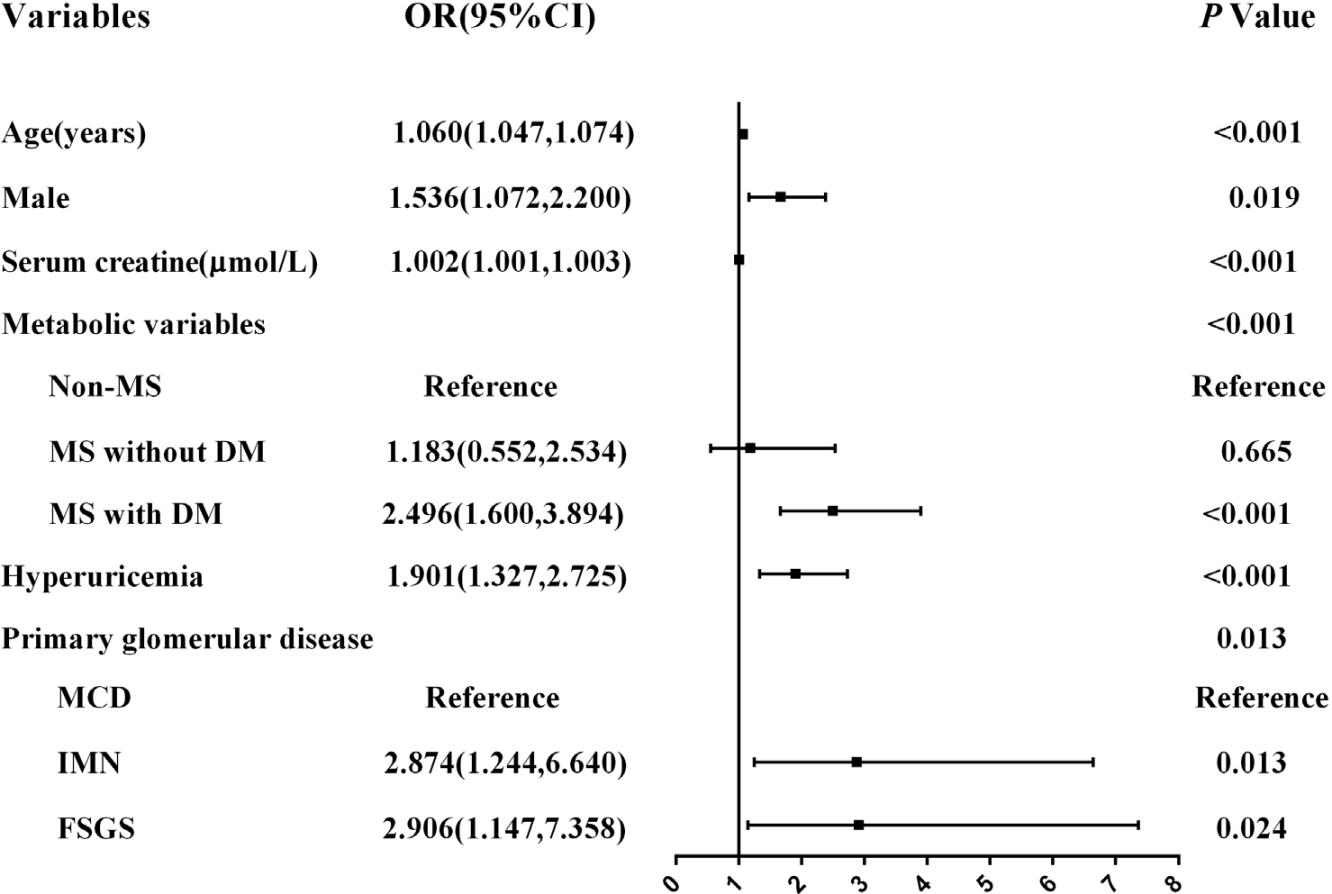
Multivariable adjusted ORs for the associations between metabolic syndrome and cardiovascular comorbidity. CI, confidence interval; DM, diabetes mellitus; FSGS, focal segmental glomerulosclerosis; IMN, idiopathic membranous nephropathy; MCD, minimal change disease; MS, metabolic syndrome; OR, odds ratio

The risk factors for cardiovascular comorbidity were evaluated with logistic regression analysis. Older age, male, higher level of serum creatine and proteinuria, lower level of eGFR, hyperuricemia, IMN, FSGS, MS, and concomitant DM were related to an increased risk of cardiovascular comorbidity in the univariate model. Higher serum albumin was protective factors for cardiovascular comorbidity in PGD (Table 2). After adjusting for multiple variables with statistical significance in univariate logistic regression, older age (OR: 1.060, 95% CI: 1.047‐1.074, *P* < .001), male (OR: 1.536, 95% CI: 1.072‐2.200, *P* = .019), higher level of serum creatine (OR: 1.002, 95% CI: 1.001‐1.003, *P* < .001) and patients with MS and concomitant DM (OR: 2.496, 95% CI: 1.600‐3.894, *P* < .001), hyperuricemia (OR: 1.901, 95% CI: 1.327‐2.725, *P* < .001), IMN (OR: 2.874, 95% CI: 1.244‐6.640, *P* < .001), FSGS (OR: 2.906, 95% CI: 1.147‐7.358, *P* < .001) remained independent risk factors for cardiovascular comorbidity in PGD (Figure 3).

**TABLE 2:** Univariate logistic regression analysis of cardiovascular comorbidity in patients with primary glomerular diseases

Variables	95% CI	OR	*P* value
Age (years)	1.062, 1.086	1.074	<.001
Male	1.393, 2.716	1.945	<.001
Serum creatine	1.001, 1.003	1.002	<.001
Serum albumin	0.964, 0.993	0.979	.004
Proteinuria (g/24 h)			<.001
<1	Reference	Reference	Reference
1‐3.5	1.021, 2.755	1.677	.041
≥3.5	1.778, 4.360	2.784	<.001
Hyperuricemia	1.590, 3.050	2.202	<.001
Microscopic hematuria	0.352, 2.684	0.972	.956
Metabolic variables			<.001
Non‐MS	Reference	Reference	Reference
MS + non‐DM	0.998, 4.060	2.013	.051
MS + DM	4.257, 9.697	6.425	<.001
Anemia	0.956, 2.415	1.519	.077
eGFR			<.001
≥90 mL/min/1.73 m^2^	Reference	Reference	Reference
60‐89 mL/min/1.73 m^2^	1.040, 2.838	1.718	.034
30‐59 mL/min/1.73 m^2^	1.571, 4.078	2.531	<.001
15‐29 mL/min/1.73 m^2^	2.980, 8.036	4.893	<.001
<15 mL/min/1.73 m^2^	3.291, 10.350	5.836	<.001
Pathological type			<.001
MCD	Reference	Reference	Reference
IgA	0.549, 2.813	1.242	.603
IMN	2.311, 11.014	5.046	<.001
FSGS	1.965, 11.203	4.691	.001
MsPGN	1.092, 17.742	4.402	.037
Other	0.913, 5.739	2.289	.077

Abbreviations: DM, diabetes mellitus; eGFR, estimated glomerular filtration rate; FSGS, focal segmental glomerulosclerosis; IgAN, IgA nephropathy; IMN, idiopathic membranous nephropathy; MCD, minimal change disease; MS, metabolic syndrome; MsPGN, mesangial proliferative glomerulonephritis.

## 4 DISCUSSION

To the best of our knowledge, this is the first study identifying the association of MS and concomitant DM with cardiovascular comorbidity among PGD population. MS and concomitant DM were identified as independent risk factor for cardiovascular comorbidity in PGD. We also identified IMN and FSGS, two common pathological subtypes of PGD, as independent predictors for cardiovascular comorbidity risk.

Cardiovascular diseases had become one of the leading causes of death in general population. According to the Global Burden of Disease Study, deaths from cardiovascular disease increased by 14.5% between 2006 and 2016.^15^ In patients with CKD, cardiovascular disease is the leading cause of death, which is closely correlated with decreasing eGFR.^16^ As one of the most common causes for CKD, PGD was closely associated with increased cardiovascular comorbidity. Due to the glomerular leakage of albuminuria, PGD were often accompanied with metabolic disorders and the development of MS, which was another cardiovascular disease (CVD)‐related risk factor. Although there were a variety of definitions for MS, it referred to the clustering of cardiovascular risk factors, including dyslipidemia, elevated blood pressure, and impaired glucose tolerance.^17^A meta‐analysis included 37 studies had detected that patients with MS had a relative risk of cardiovascular events and death of 1.78 (95% CI 1.58‐2.00) compared with those without MS.^18^ DM had also been recognized as an independent risk factor for cardiovascular disease. Patients with DM have fourfold increases in chances of having cardiovascular disease compared to subjects without DM.^19^ Both MS and DM are thought to be associated with an increased risk for cardiovascular events. However, the coexistence of multiple variables in DM patients such as diabetic duration and glycemic control status may confound the impact of MS on the cardiovascular risk. Whether MS coexisted with DM or not would increase risk for cardiovascular disease in PGD had not been explored before. Previous studies had found that patients with MS and DM presented with more cardiovascular risk factors such as hypertension and hyperlipidemia compared to MS alone.^20^ According to the baseline characteristics in our research, MS was more likely to occur in elder PGD patients and coexisted with higher levels of serum creatine, proteinuria, uric acid and lower levels of eGFR. These findings suggested that MS was associated with severe renal insufficiency and metabolic disorder in PGD. In addition, age, proteinuria, uric acid, triglyceride, proportion of hypertension, hyperuricemia, and hyperlipidemia were significantly higher in subjects with MS and DM compared to MS alone. Therefore, DM with MS may be a marker of high risk for future cardiovascular events at baseline rather than MS alone.

The other important finding of our study is that patients with MS and DM presented with higher prevalence of cardiovascular comorbidity compared with non‐DM stratified by eGFR and pathological types. Logistic regression analysis showed that co‐occurrence of MS with DM was associated with an increased risk of cardiovascular comorbidity in PGD. The joint impact of MS and DM on cardiovascular comorbidity in PGD population is incompletely understood, but several possible mechanisms may contribute to it^21^, ^22^, ^23^ First, MS, DM, proteinuria and renal insufficiency in PGD often coexist with other cardiovascular risk factors such as hypertension and hyperlipidemia, which may contribute to the occurrence of cardiovascular comorbidity. Furthermore, MS, DM, proteinuria and renal insufficiency in PGD may accelerate the progression of endothelial dysfunction, atherosclerosis, inflammation, which play a major role in the pathogenesis of cardiovascular events. Thus, MS, DM coupled with renal insufficiency and proteinuria may contribute to the increased risk of cardiovascular disease in subjects with PGD. However, we did not observe an association between MS without DM and cardiovascular comorbidity after adjustment for other predictors. Several researches had also found that MS without DM was not a predictor for cardiovascular events.^24^, ^25^ Although some reviews^1^ had concluded that MS was associated with a significantly increased risk of cardiovascular comorbidity, these analyses included many MS patients with concomitant DM and could not determine whether MS represented an independent risk beyond its component factors.^25^

In several retrospective and prospective studies, MS had been significantly associated with the development and progression of CKD^26^, ^27^, ^28^ Previous study had found that MS occurred in 30.5% of stages 4 and 5 CKD patients.^6^ However, in our study, only 8.5% of PGD patients were coexisted with MS. This is probably because 84.3% PGD patients were in CKD stage 1 to 3, which were regarded as mild CKD. Another important finding of our study is that older age, male, higher serum creatine, hyperuricemia, IMN and FSGS were independent predictors for cardiovascular comorbidity in PGD. Older age, male, higher level of serum creatine and hyperuricemia^29^, ^30^, ^31^, ^32^ had been previously proved to be associated with increased cardiovascular risk. Furthermore, patients with IMN and FSGS are at higher risk of cardiovascular comorbidity compared with MCD and IgAN. The finding of higher prevalence of cardiovascular comorbidity in IMN was consistent with the recent published retrospective study.^33^ However, higher risk of cardiovascular comorbidity in FSGS had never been investigated before. It is conceivable that higher cardiovascular risk in IMN and FSGS may be more directly related to the nephrotic syndrome, which was more common in IMN and FSGS compared with other pathological types in PGD. Furthermore, although IgAN is the most common PGD in China^,^ ^34^ we did not observe higher cardiovascular risk in IgAN compared with other pathological types. This may be associated with complicated clinical manifestations and individual variations, especially quantitative proteinuria in IgAN. We had detected that the prevalence of cardiovascular comorbidity in IgAN with quantitative proteinuria greater than 1.0 g/24 hours was higher than those with quantitative proteinuria less than 1.0 g/24 hours (2.94% vs 1.45%, *χ* ^2^ = 4.110, *P* = .043). Therefore, for IgAN with massive proteinuria, cardiovascular comorbidity risk still deserves equal attention during follow‐up. In addition, although proteinuria was a proved risk factor for cardiovascular diseases, we did not demonstrate it in PGD population. Distinct quantitative proteinuria and clinical manifestation were found in different pathological types of PGD, which may confound the impact of proteinuria on cardiovascular risks in this study.

Importantly, our study for the first time explored the joint association between MS accompanying DM and cardiovascular comorbidity in large Chinese PGD population. However, our study still had several limitations. Firstly, due to the retrospective nature and the lack of long‐term follow‐up, we could not explore that the presence of MS and concomitant DM at baseline were risk factor for developing subsequent cardiovascular diseases in PGD. In addition, there were several definitions of MS including world health organization (WHO) and the NCEP adult treatment panel (ATP) III.^35^ The definition of MS we applied^10^ did not include elevated waist due to lack of waist circumference index, which may underestimate the prevalence of MS in PGD population.

## 5 CONCLUSIONS

In PGD patients, MS and concomitant DM were associated with an increased risk for cardiovascular comorbidity. IMN and FSGS were also independent predictors for higher cardiovascular risk in PGD. The detection, prevention, and treatment of MS and concomitant DM should become an important approach for the reduction of cardiovascular burden in the PGD population.

## CONFLICT OF INTEREST

The authors declare no potential conflict of interests.

## Supporting information


**Table S1**: Clinical baseline characteristics in non‐cardiovascular comorbidity and cardiovascular comorbidity patients.
