# Supplementary material for: Metabolic syndrome and concomitant diabetes mellitus are associated with higher risk of cardiovascular comorbidity in patients with primary glomerular diseases: A retrospective observational study
Source: Clin Cardiol. 2020 May 29;43(9):949–56. doi: 10.1002/clc.23388 (PMC8101350; doi:10.1002/clc.23388)
Supplement: Supplementary file 1 — Table S1: Clinical baseline characteristics in non‐cardiovascular comorbidity and cardiovascular comorbidity patients. [file CLC-43-949-s001.docx]

**Supplementary Table 1. Clinical baseline characteristics in non-cardiovascular comorbidity and cardiovascular comorbidity patients.**

|  | Non-Cardiovascular comorbidity  (n=3,458) | Cardiovascular comorbidity  (n=164) | *P*-value |
| --- | --- | --- | --- |
| Male (n, %) | 1,793 (51.9) | 111 (67.7) | <0.001 |
| Age (years) | 40±15 | 57±13 | <0.001 |
| BMI (kg/m^2^) | 23.5±4.0 | 24.4±3.1 | 0.087 |
| Serum creatine (μmol/L) | 95.0(69.8,146.1) | 140.1(85.3,255.6) | <0.001 |
| eGFR (ml/min/1.73m^2^) | 74.4±36.1 | 50.9±33.2 | <0.001 |
| Serum albumin (g/L) | 27.9±10.4 | 25.5±9.2 | <0.001 |
| Proteinuria (g/24h) | 2.3(0.8,5.7) | 4.6(1.7,9.2) | <0.001 |
| Uric Acid (μmol/L) | 397.1±173.0 | 446.7±152.0 | <0.001 |
| Hemoglobin (g/L) | 130.2±22.0 | 124.6±19.8 | 0.001 |
| Cholesterol (mmol/L) | 6.9±3.5 | 7.3±3.2 | 0.098 |
| Triglyceride (mmol/L) | 2.4±2.1 | 2.9±2.0 | 0.004 |
| HDL-C (mmol/L) | 1.3±0.6 | 1.3±0.5 | 0.198 |
| LDL-C (mmol/L) | 4.0±2.2 | 4.5±2.2 | 0.004 |
| Hypertension (n, %) | 1,111(32.1) | 115(70.1) | <0.001 |
| Diabetes (n, %) | 221(6.4) | 41(25.0) | <0.001 |
| Hyperuricemia (n, %) | 748(21.6) | 62(37.8) | <0.001 |
| Hyperlipidemia (n, %) | 298(8.6) | 22(13.4) | 0.034 |

BMI, Body mass index; eGFR, estimated Glomerular Filtration Rate; HDL-C, high density lipoprotein-cholesterol; LDL-C, Low density lipoprotein-cholesterol
